# Manifestações Cardiovasculares na População Pediátrica com COVID-19: Qual a Real Importância?

**DOI:** 10.36660/abc.20210835

**Published:** 2021-11-01

**Affiliations:** 1 Hospital Evangélico de Cachoeiro de Itapemirim Cachoeiro de Itapemirim ES Brasil Hospital Evangélico de Cachoeiro de Itapemirim, Cachoeiro de Itapemirim, ES – Brasil; 2 Universidade de São Paulo Faculdade de Medicina São Paulo SP Brasil Faculdade de Medicina da Universidade de São Paulo, São Paulo, SP – Brasil; 3 Fundação Técnico Educacional Souza Marques Rio de Janeiro RJ Brasil Fundação Técnico Educacional Souza Marques, Rio de Janeiro, RJ – Brasil

**Keywords:** COVID-19, Coronavírus, Síndrome Inflamatória, Doenças Cardiovasculares, Criança, Biomarcadores, Ecocardiografia/métodos, Strain

Embora as manifestações da COVID-19 sejam leves em crianças, a síndrome inflamatória multissistêmica (SIM) pode ocorrer em 0,6 dos casos. A SIM pediátrica (SIM-P) já foi bem definida pela Organização Mundial da Saúde (OMS) e é caracterizada por hiperinflamação com tempestade de citocinas e níveis elevados de marcadores de lesão do miocárdio, com envolvimento de um ou mais órgãos dos sistemas cardíaco, renal, respiratório, gastrointestinal ou neurológico.^1^

A combinação do momento de ocorrência da SIM-P com sorologia positiva e PCR negativo na maioria dos pacientes sugere que a SIM-P seja mais uma complicação pós-infecciosa (até seis semanas após o insulto), mediada pelo sistema imune, que uma complicação da infecção aguda. Acredita-se que a fisiopatologia da SIM-P seja devido a uma resposta imune exacerbada em uma criança geneticamente susceptível. Os sintomas de SIM-P podem se sobrepor aos sintomas de doença de Kawasaki, síndrome do choque tóxico, síndrome de ativação macrofágica, sepse bacteriana, e síndrome de liberação de citocinas (“tempestade de citocinas”). A tempestade de citocinas é caracterizada por febre persistente, com níveis elevados de marcadores inflamatórios e citocinas pró-inflamatórias, tais como a interleucina.^2^ Há evidências crescentes sobre o envolvimento cardiovascular na COVID-19 e na SIM-P.^3,4^

Em um recente estudo multicêntrico europeu, Valverde et al.^5^ demonstraram manifestações cardiovasculares agudas em 286 crianças com idade média de 8,4 anos (3,8 a 12,4 anos), cujas complicações mais frequentes foram choque, arritmia cardíaca, efusão pericárdica, dilatação da artéria coronária, e elevação da troponina em 93% dos casos. Foi registrada uma morte por arritmia ventricular e havia um paciente na lista de transplante cardíaco.^5^ Em outro estudo com 186 pacientes com SIM-P em 26 estados americanos, comprometimento cardíaco foi observado em 80% dos pacientes, e 33% desses apresentaram fração de ejeção ventricular esquerda (FEVE) menor que 55%, e 5% apresentaram FEVE menor que 30%. Níveis aumentados de troponina e peptídeo natriurético tipo-B (BNP) foram observados em 50% e 73% dos pacientes, respectivamente, efusão pericárdica em 26%, arritmia cardíaca em 12%, e envolvimento cardíaco em 8%.^6^ Um estudo conduzido na América Latina, com participação de centros brasileiros, mostrou que crianças com COVID-19 e envolvimento cardiovascular tiveram apresentação clínica mais grave, com maiores alterações laboratoriais, instabilidade hemodinâmica, necessidade de drogas vasoativas e maior número de internação em unidade de terapia intensiva.^7^

Os mecanismos potenciais de lesão miocárdica na COVID-19 variam desde cardiotoxicidade viral direta, conforme relatado pelo grupo do Instituto da Criança do HCFMUSP-São Paulo-Brasil em uma criança de 11 anos de idade com SIM-P que desenvolveu taquicardia ventricular indo a óbito dentro de 28 horas da admissão, e teve partículas virais detectadas no tecido cardíaco,^8^ até outros fatores tais como microtrombose, disfunção microvascular, estado hiperinflamatório, hipoxemia, aumento da demanda metabólica e hipotensão.^9^

A ecocardiografia tem se destacado como um método robusto tanto para o diagnóstico como para o seguimento de pacientes pediátricos com COVID-19 e já foi indexada nas diretrizes clínicas para SIM-P.^2^ Vários parâmetros são avaliados pela ecocardiografia, tais como função sistólica e diastólica, efusão pericárdica, alterações valvares, comprometimento da artéria coronária incluindo hiperecogenicidade, irregularidades da parede, dilatação e aneurismas avaliados com as medidas dos diâmetros das artérias coronárias e analisados pelos z-scores. Em várias séries publicadas durante a pandemia, o envolvimento da artéria coronária em pacientes com SIM-P foi observado entre 8 e 36%, provavelmente devido à disfunção endotelial associada com tempestade de citocinas causada pelo SARS-CoV-2.^2^

No estudo intitulado “O Coração de Pacientes Pediátricos com COVID-19: Novos Insights a Partir de um Estudo Ecocardiográfico Sistemático em um Hospital Terciário no Brasil”,^10^ os autores avaliaram retrospectivamente 48 pacientes pediátricos, 73% com doenças pré-existentes e 41,7% com SIM-P. Foram realizadas avaliações ecocardiográficas padronizadas, com adequadas variabilidades intraobservador e entre observadores. Anormalidades ecocardiográficas foram significativamente associadas com SIM-P, internação e maior permanência na unidade de terapia intensiva pediátrica, disfunção de múltiplos órgãos, suporte ventilatório/vasoativo e morte. Observou-se uma interessante correlação estatisticamente significativa dos achados ecocardiográficos com alterações nos marcadores inflamatórios e lesão miocárdica. Pacientes com disfunção do ventrículo esquerdo apresentaram níveis mais elevados de D-dímero, proteína C reativa, ferritina e troponina, aqueles com disfunção ventricular direita apresentaram níveis mais elevados de D-dímero e proteína C-reativa e aqueles com anormalidades na artéria coronária apresentaram níveis mais elevados de D-dímero somente. Devido a esses achados, os autores destacam a importância da imunotrombose na SIM-P e levantam a hipótese de se bloquear a cascata de coagulação para diminuir a resposta inflamatória, enfatizando as propriedades não anticoagulantes da heparina, tais como inibição da quimiotaxia de neutrófilos e migração de leucócitos, com consequente diminuição dos biomarcadores inflamatórios. O uso da heparina, além da sua função antitrombótica, melhoraria a terapia anti-inflamatória juntamente com a imunomodulação realizada pela imunoglobulina humana endovenosa, corticoides e agentes imunobiológicos em pacientes com SIM-P. ^11^

**Figura 1:**
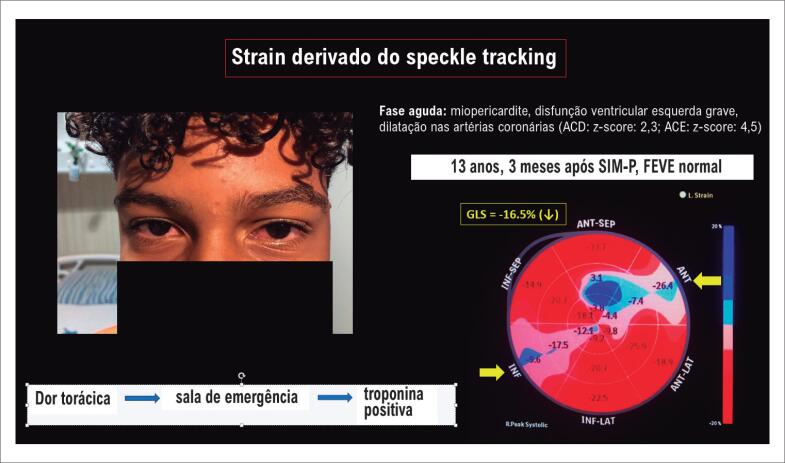
Adolescente de 13 anos com febre e dor torácica, apresentou hiperemia conjuntival e troponina positiva. Ecocardiograma na fase aguda apresentou miopericardite, disfunção ventricular esquerda, dilatação da artéria coronária direita (ACD) (z-score 2,3) e pequeno aneurisma na artéria coronária esquerda (ACE) (z-score: 4,5). Após três meses da síndrome inflamatória multissistêmica, apesar da normalização da função do ventrículo esquerdo (ao ecocardiograma bidimensional), o paciente ainda apresentava deformação miocárdica anormal ao estudo com strain derivado do specke tracking (setas amarelas no bull's eye - post).

Outra importante ferramenta que agrega valor à ecocardiografia é o uso da deformação miocárdica (*strain*) avaliada pela técnica de *speckle tracking* em pacientes pediátricos com SIM-P tanto na fase aguda como no acompanhamento (Figura 1). Matsubara et al.^12^ demonstraram que a disfunção cardíaca sistólica e diastólica pode ocorrer com frequência, provavelmente devido à uma condição similar à miocardite. Mesmo em pacientes com fração de ejeção preservada, foram detectadas mudanças sutis na deformação do miocárdio, sugerindo uma disfunção subclínica do miocárdio. Além disso, esses autores demonstraram que mesmo os pacientes cujos valores de fração de ejeção foram normalizados continuaram com alterações diastólicas ventriculares na avaliação ecocardiográfica pelo *speckle tracking* ao longo do tempo, reforçando a necessidade do acompanhamento de pacientes com SIM-P e realização de exames complementares (troponina, peptídeo natriurético cerebral, eletrocardiograma, Holter, ecocardiograma, e ressonância magnética em alguns casos).

As manifestações cardiovasculares da COVID-19 são comuns em pacientes com SIM-P e podem levar a alta morbidade e consequente morte. O entendimento sobre a síndrome relacionada à SARS-CoV-2 na população pediátrica é crescente. Todo conhecimento acumulado até o momento reflete as evidências disponíveis atualmente, juntamente com a opinião de especialistas. Há muito o que se aprender acerca da SIM-P na COVID-19 visando um melhor diagnóstico, tratamento e acompanhamento desses pacientes.
