# Supplementary material for: Manifestações Cardiovasculares na População Pediátrica com COVID-19: Qual a Real Importância?
Source: Arq Bras Cardiol. 2021 Nov 1;117(5):965–7. [Article in Portuguese] doi: 10.36660/abc.20210835 (PMC8682094; doi:10.36660/abc.20210835)
Supplement: Translation

# Translation: Cardiovascular Manifestations in the Pediatric Population with COVID-19, What is the Real Relevance?

**Affiliations:** 1 Cachoeiro de Itapemirim ES Brazil Hospital Evangélico de Cachoeiro de Itapemirim, Cachoeiro de Itapemirim, ES – Brazil; 2 São Paulo SP Brazil Faculdade de Medicina da Universidade de São Paulo, São Paulo, SP – Brazil; 3 Rio de Janeiro RJ Brazil Fundação Técnico Educacional Souza Marques, Rio de Janeiro, RJ – Brazil

**Keywords:** COVID-19, Coronavirus, Inflammatory Syndrome, Cardiovascular Dseases, Child, Biomarkers, Echocardiography/methods, Strain

Although COVID-19 usually has mild manifestations in children, multisystem inflammatory syndrome (MIS) may occur in 0.6% of the cases. MIS in children (MIS-C), already well defined by the World Health Organization (WHO), is characterized by hyperinflammation with cytokine storm and high levels of myocardial injury markers, with involvement of one or more organs of the cardiac, renal, respiratory, gastrointestinal or neurological systems.^1^

The combination of the timing of MIS-C cases with positive serology and negative PCR in most patients suggests that MIS-C is a post-infectious (up to six weeks after the insult), immune-mediated complication rather than of the acute infection. The pathophysiology of MIS-C is thought to be due to a hyperimmune response to the virus in a genetically susceptible child. The symptoms of MIS-C can overlap with those of Kawasaki disease, toxic shock syndrome, macrophage activation syndrome, bacterial sepsis, and cytokine release syndrome (“cytokine storm”). Cytokine storm is characterized by persistent fever, and markedly elevated inflammatory markers and pro-inflammatory cytokines such as interleukin.^2^ There increasing evidence on cardiovascular involvement in COVID-19 and MIS-C.^3,4^

In a recent European multicenter study, Valverde et al.^5^ demonstrated acute cardiovascular manifestations in 286 children with an average age of 8.4 years (3.8 to 12.4 years), whose most common complications were shock, cardiac arrhythmia, pericardial effusion, coronary dilation, and troponin elevation in 93% of the cases. There were one death due to ventricular arrhythmia and one patient listed for heart transplantation.^5^ In another study with 186 patients with MIS-C in 26 American states, cardiovascular involvement was observed in 80% of patients, and 33% of these had left ventricular ejection fraction (LVEF) less than 55% and 5% had LVEF less than 30%. Increased troponin and B-type natriuretic peptide (BNP) e levels were found in 50% and 73% of patients, respectively, pericardial effusion in 26%, cardiac arrhythmia in 12%, and coronary involvement in 8%.^6^ A Latin American study^7^ that also included the participation of Brazilian centers showed that children with COVID-19 with cardiovascular involvement had more severe clinical presentation, more laboratory abnormalities, greater hemodynamic instability and the greater need for vasoactive drugs and intensive care unit admission, when compared to those without cardiovascular involvement.^7^

The potential mechanisms of myocardial injury in COVID-19 range from direct viral cardiotoxicity, as first reported by the group of the Instituto da Criança of HCFMUSP - São Paulo - Brazil, about a case of an 11-year-old child affected by MIS-C, who developed ventricular tachycardia and died within 28 hours of admission, and had viral particles detected in the cardiac tissue,^8^ to several other factors such as microthrombosis, microvascular dysfunction, endothelial injury, hyperinflammatory state, hypoxemia, increased metabolic demand and hypotension.^9^

Echocardiography has emerged as a robust tool both in diagnosis and in clinical follow-up in the pediatric population with COVID-19 and has already been indexed in the clinical guideline for MIS-C.^2^ Several parameters are evaluated by echocardiography, such as left ventricular systolic and diastolic function, pericardial effusion, valvular alterations, coronary involvement such as increased hyperechogenicity, parietal irregularities, dilation, and aneurysms by measurement of the of coronary artery diameter and analysis using z-score. In several series published during the pandemic, coronary involvement in MIS-C occurred from 8 to 36%, probably due to endothelial dysfunction associated with cytokine storm caused by SARS-CoV-2.^2^

In the study “The Heart of Pediatric Patients with COVID-19: New Insights from a Systematic Echocardiographic Study in a Tertiary Hospital in Brazil”,^10^ the authors retrospectively evaluated 48 pediatric patients, 73% with pre-existing diseases and 41.7% with MIS-C. Standardized echocardiographic assessments were performed with adequate intraobserver and interobserver variability. Echocardiographic abnormalities were significantly associated with MIS-C, admission and longer stay in the pediatric intensive care unit, multiple organ dysfunction, ventilatory/vasoactive support, and death. There was an interesting, statistically significant correlation of echocardiographic findings with changes in inflammatory markers and myocardial injury. Patients with left ventricular dysfunction had higher levels of D-dimer, C-reactive protein, ferritin and troponin; those with right ventricular dysfunction had higher levels of D-dimer and C-reactive protein; and those with coronary abnormalities had higher levels of D-dimer only. Due to these findings, the authors highlight the importance of immunothrombosis in MIS-C and raise the hypothesis of blocking the coagulation cascade to decrease the inflammatory response, emphasizing the non-anticoagulant properties of heparin, such as the inhibition of neutrophil chemotaxis and migration of leukocytes, with a consequent decrease in inflammatory biomarkers. The use of heparin, in addition to its antithrombotic function, would enhance the anti-inflammatory therapy together with the immunomodulation performed by intravenous human immunoglobulin, corticosteroids and immunobiological agents in patients with MIS-C.^11^

Another important tool that has added value to echocardiography is the use of the strain assessed by the speckle tracking technique in pediatric patients with SIM-P both in the acute phase and in the follow-up (Figure 1). Matsubara et al.^12^ demonstrated that systolic and diastolic cardiac dysfunctions might occur frequently, probably due to the presence of a state similar to myocarditis. Even in patients with preserved ejection fraction, subtle changes in myocardial deformation were detected, suggesting subclinical myocardial dysfunction. In addition, these authors demonstrated that even patients whose ejection fraction was normalized, remained with ventricular diastolic changes in the echocardiographic assessment by speckle tracking over time, reinforcing the need for the follow-up of patients with MIS-C with complementary exams (troponin, BNP, electrocardiogram, Holter, echocardiogram and magnetic resonance in some cases).

**Figure 1:** A 13-year-old teenager presenting with fever and chest pain, showed conjunctival hyperemia and positive troponin. Acute-phase echocardiogram revealed myopericarditis, left ventricular dysfunction, right coronary artery (RCA) dilation (z-score: 2.3) and small left main coronary artery (LMCA) aneurysm (z-score: 4.5). After three months of multisystem inflammatory syndrome, despite normalization of the left ventricular function (by two-dimensional echocardiogram), the patient still had abnormal myocardial strain (yellow arrows) and decreased global longitudinal strain (bull's eye-plot).

The cardiovascular manifestations of COVID-19 are frequent in patients with MIS-C and can lead to high morbidity and consequent deaths. The understanding of the SARS-CoV-2-related syndrome in the pediatric population is growing. All knowledge gained to date reflects the current evidence available together with expert opinion. There is a lot to learn about MIS-C in COVID-19, towards better diagnosis, treatment and follow-up of these patients.
